# Individual dynamic prediction and prognostic analysis for long-term allograft survival after kidney transplantation

**DOI:** 10.1186/s12882-022-02996-0

**Published:** 2022-11-07

**Authors:** Baoyi Huang, Mingli Huang, Chengfeng Zhang, Zhiyin Yu, Yawen Hou, Yun Miao, Zheng Chen

**Affiliations:** 1 Department of Biostatistics, School of Public Health (Guangdong Provincial Key Laboratory of Tropical Disease Research), Southern Medical University, Guangzhou, 510515 China; 2 Department of Transplantation, Nanfang Hospital, Southern Medical University, Guangzhou, 510515 China; 3 Department of Statistics, School of Economics, Jinan University, Guangzhou, 510632 China

**Keywords:** Dynamic prediction, Kidney transplantation, Longitudinal biomarkers, Precise medicine, Individual prediction

## Abstract

**Background:**

Predicting allograft survival is vital for efficient transplant success. With dynamic changes in patient conditions, clinical indicators may change longitudinally, and doctors’ judgments may be highly variable. It is necessary to establish a dynamic model to precisely predict the individual risk/survival of new allografts.

**Methods:**

The follow-up data of 407 patients were obtained from a renal allograft failure study. We introduced a landmarking-based dynamic Cox model that incorporated baseline values (age at transplantation, sex, weight) and longitudinal changes (glomerular filtration rate, proteinuria, hematocrit). Model performance was evaluated using Harrell’s C-index and the Brier score.

**Results:**

Six predictors were included in our analysis. The Kaplan–Meier estimates of survival at baseline showed an overall 5-year survival rate of 87.2%. The dynamic Cox model showed the individual survival prediction with more accuracy at different time points (for the 5-year survival prediction, the C-index = 0.789 and Brier score = 0.065 for the average of all time points) than the static Cox model at baseline (C-index = 0.558, Brier score = 0.095). Longitudinal covariate prognostic analysis (with time-varying effects) was performed.

**Conclusions:**

The dynamic Cox model can utilize clinical follow-up data, including longitudinal patient information. Dynamic prediction and prognostic analysis can be used to provide evidence and a reference to better guide clinical decision-making for applying early treatment to patients at high risk.

**Supplementary Information:**

The online version contains supplementary material available at 10.1186/s12882-022-02996-0.

## Introduction

Chronic kidney disease (CKD) is a major public health issue with increasing attention and prevalence worldwide [1]. Kidney transplantation is well recognized as the best treatment option for patients with end-stage renal disease [2]. However, there are still a substantial number of kidney transplantation failures due to various causes, such as rejection, infection and recurrence of glomerulonephritis. Failure after transplantation is a burden on the transplant system, health care system, and even the patient’s quality of life [3]. Therefore, predicting renal allograft survival is vital for efficient transplant success.

During long-term performance after kidney transplantation, a great deal of information, ranging from lab results such as glomerular filtration rate (GFR) to patients’ conditions such as blood pressure, is measured repeatedly in patients over time during observational studies and clinical trials or simply as patients undergo routine monitoring [4]. The loss of kidney function (renal allograft failure) is often defined as an interesting end point in clinical research [5, 6]; thus, the time from baseline to the events of clinical interest is usually collected alongside these longitudinal data.

**Fig. 1:**
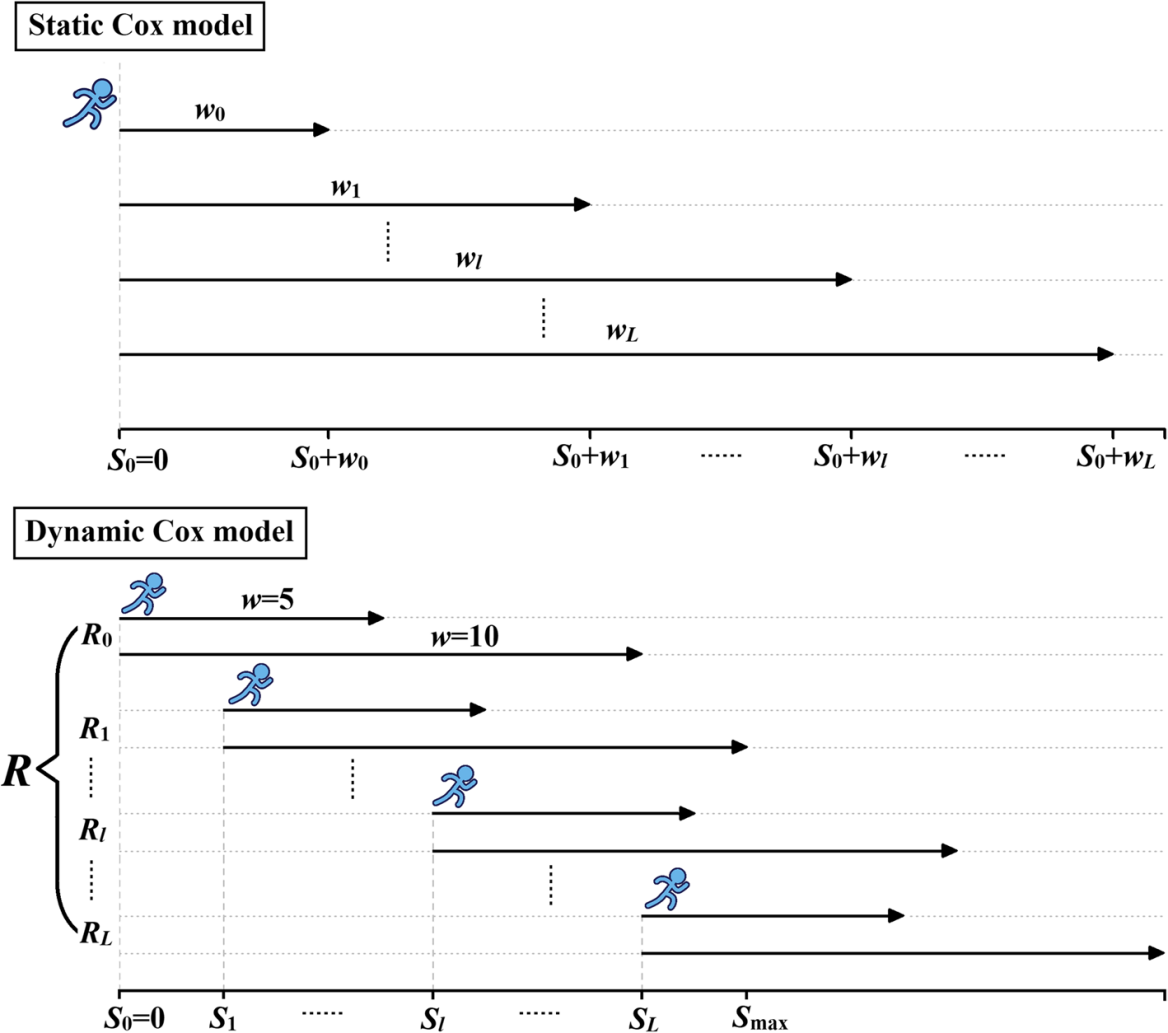
Prediction processes of the static Cox model and the dynamic Cox model

In recent years, several prediction models (e.g., the Cox regression model) for post-kidney transplantation have been developed and validated [7–15]. However, most studies were limited to making static predictions at a fixed time point and with fixed covariates, such as the “static prediction” shown in Fig. 1. With the follow-up of kidney transplant patients over time, it is necessary to assess various biomarkers at periodic follow-up visits and to ensure that patients’ conditions are stable. These biomarkers are usually defined as longitudinal time-dependent covariates whose values may change over time [16]. For example, the GFR is a clinical sign of renal function for patients in whom it is measured multiple times. An increase in the GFR means that the patient’s renal function is gradually returning to normal. Therefore, longitudinal information should be used to predict the risk of kidney transplant failure.

With the development of precision medicine, building a predictive model with strong performance to achieve accurate predictions for individuals is one essential aspect [7]. Prediction models for precision medicine can be used to guide clinical decision-making on diagnosis and treatment, which has important clinical implications for personalized kidney transplant monitoring based on the risk of transplant failure. For example, anticipating therapy for patients with a high risk of losing renal function is key to improving allograft survival. Thus, the predicted risk of allograft failure determines who should receive early treatment and when it should be applied. Therefore, the longitudinal information obtained during follow-ups should be used to predict patient survival and aid in clinical decision-making, which enables more precise and dynamic individual predictions.

Therefore, we need to build a “dynamic” prediction model. The method of adding patients’ changing information (longitudinal time-dependent covariates) to the prediction model and updating the prediction results at different prediction times (both at the baseline and at later time points) is called “dynamic prediction” [17, 18]. As shown in Fig. 1, dynamic prediction can yield the survival probabilities of patients in the next *w* years (*w* = 5 or *w* = 10) at different prediction time points.

At present, one of the commonly used methods to achieve dynamic prediction is the landmarking method. This method constructs a prediction dataset with the information of patients at risk at specific landmark times *s*_*l*_. The Cox model employing the landmarking method can utilize updated survival data to construct a prediction model that spans multiple landmark time points to obtain a dynamic survival probability. Therefore, such a Cox model can capture the development of time-dependent covariates, and this model is called the dynamic Cox model [18–20].

In this study, we introduced a dynamic Cox model based on the landmarking approach and applied it to a example data from observational studies after kidney transplantation. The dynamic change performance outcomes of the prediction model were assessed, and specific patient examples were used to illustrate how the predicted survival probabilities of new allografts change at different prediction times during follow-up.

## Materials and methods

### Example data

In a renal allograft failure study [5], 407 patients with CKD underwent kidney transplantation in the hospital of the Catholic University of Leuven in Belgium between January 1983 and August 2000 and were followed until renal allograft failure or censorship. The outcome of this analysis was overall survival, which was calculated in years as the time from transplantation to renal allograft failure. Patients whose allografts were still functioning at the last follow-up were censored. Six predictors were selected for the analysis, including age at transplantation, sex, and weight measured at baseline. The three longitudinal time-varying covariates were hematocrit, proteinuria and GFR, and these biomarkers were measured regularly during follow-ups to check the allograft condition of the patients.

### Statistical methods

Descriptive statistics are reported as the means ± standard deviations for continuous variables and the number of patients for categorical variables. A landmark analysis was performed to make dynamic predictions.

#### Landmarking approach

The basic idea of the landmarking method is to predetermine a series of meaningful time points and assess the statuses of patients at these moments. As shown in Fig. 1, we defined landmark time points *s*_*l*_ on the expected prediction interval [0, *s*_max_], where *s*_max_ is the maximum prediction time of interest, to estimate the survival of new allografts in the next *w* years (prediction window *w* = 5 or *w* = 10) at different prediction time points *s*_*l*_. For each *s*_*l*_ (*l* = 0, 1..., *L*), we selected individuals who were still at risk (still alive and undergoing follow-up) at time point *s*_*l*_ and neglected any event after *s*_*l*_ *+ w* to construct a corresponding landmark dataset *R*_*l*_.

#### Dynamic Cox model

As shown in Fig. 1, we constructed the dataset ***R*** by stacking all landmark datasets *R*_*l*_ together and fitting a Cox model on ***R***. This model had an effect function *β*(*s*_*l*_) = *β*_0_ + *β*_1_*s*_*l*_ + *β*_2_*s*_*l*_^2^ for covariates *Z* at different prediction time points *s*_*l*_, where *β*_0_, *β*_1_ and *β*_2_ are parameters, and a time function *θ*(*s*_*l*_) = *θ*_1_*s*_*l*_ + *θ*_2_*s*_*l*_^2^, where *θ*_1_ and *θ*_2_ are parameters. We built a dynamic Cox model as follows:

$$h\left(t|Z,{s}_l\right)={h}_0(t)\exp \left({Z}^{\textrm{T}}\left({s}_l\right)\cdot \beta \left({s}_l\right)+\theta \left({s}_l\right)\right),{s}_l\le t\le {s}_l+w$$

#### Predictive performance assessment

The measures used to evaluate the performance of predictive models are usually divided into discrimination and calibration measures. Harrell’s C-index is generally used for discrimination [21]. The higher the C-index is, the better the consistency, which means that the predicted survival probability is more consistent with the patient’s real survival time. The Brier score is a calibrated metric that calculates the squared difference between the probability predicted by the prediction model and the observed result [22]. A smaller Brier score indicates a higher accuracy of prediction by the model. The process of Monte Carlo cross-validation with 200 iterations is shown in Supplementary Fig. S1 and was used to avoid overfitting. Then, the average C-index and Brier score values were calculated to assess the performance of the two models.

#### Dynamic analysis

We aimed to construct a dynamic prediction model that could provide patients with dynamic survival predictions for new allografts. Compared with the static Cox model, the dynamic Cox model can update the survival of new allografts at different prediction time points. After fitting the dynamic Cox model, the predicted *w*-year dynamic survival probability at landmark time point *s*_*l*_ was calculated:

$$S\left({s}_l+w|{s}_l,Z\right)=P\left(T>{s}_l+w|T>{s}_l,Z\right)=\exp \left(-{\int}_{s_l}^{s_l+w}h\left(t|Z,{s}_l\right) dt\right)$$

To perform precise individual prediction, several patients were selected from the data, and we could dynamically predict the conditional survival probabilities and real-time survival rate of new allografts for these patients in the next *w* years.

For numeric stability, the landmark time point *s*_*l*_ was standardized using *s*_*l*_/(*s*_*L*_-*s*_0_), which ranged from 0 to 1, to calculate the dynamic hazard ratio (HR). Then, the *w*-year dynamic HR could be calculated as follows:

$${HR}^w\left({s}_l\right)=\exp \left({\beta}_0+{\beta}_1\times \left({s}_l/\left({s}_L-{s}_0\right)\right)+{\beta}_2\times {\left({s}_l/\left({s}_L-{s}_0\right)\right)}^2\right).$$

## Results

### Data description

All 407 patients were included in the analysis. The median follow-up time was 11.86 years (range: 1.04 ~ 19.22 years). The baseline characteristics of the study population are depicted in Table 1. Supplementary Fig. S2 shows the Kaplan–Meier estimates of survival at prediction time point *s* = 0 (baseline), with an overall 5-year survival rate of 87.2% (95% confidence intervals: 84.0% ~ 90.5%) and an overall 10-year survival rate of 77.9% (95% confidence intervals: 74.0% ~ 82.0%).

**Table 1:** Baseline characteristics and results from the static Cox model and the dynamic Cox model (*w*=5)

Variables	Mean ± SD	Static Cox model			Dynamic Cox model (***w*** = 5)			
		Coef	SE (coef)	***P*** value	Time function	Coef	SE (coef)	***P*** value
Age	4.160 ± 1.278	−0.212	0.079	0.007	1	−0.447	0.023	< 0.001
Sex	220 (male)	0.206	0.210	0.325	\	\	\	\
Weight	6.381 ± 1.142	0.126	0.090	0.162	1	0.603	0.026	< 0.001
Hematocrit	2.951 ± 0.620	0.148	0.146	0.310	1	0.256	0.083	0.143
	\	\	\	\	***s***_***l***_ **/** 10	−1.498	0.165	< 0.001
GFR	0.891 ± 0.549	0.133	0.128	0.298	1	−0.375	0.028	< 0.001
	\	\	\	\	***s***_***l***_ **/** 10	− 0.545	0.065	0.015
Proteinuria	2.409 ± 3.583	−0.032	0.041	0.433	1	−0.182	0.039	0.053
	\	\	\	\	***s***_***l***_ **/** 10	1.948	0.171	< 0.001
	\	\	\	\	(***s***_***l***_ **/** 10)^2^	−1.902	0.157	< 0.001
*θ*(*s*)	\	\	\	\	***s***_***l***_ **/** 10	8.444	0.703	< 0.001
	\	\	\	\	(***s***_***l***_ **/** 10)^2^	−1.957	0.454	< 0.001

Coef, coefficient; SE, standard error; age, age at transplantation (per 10 years); sex (male = 0, female = 1); weight (per 10 kg); hematocrit (per 0.1%); GFR, glomerular filtration rate (per 10 ml/min); proteinuria (per 1 g/24 hours)

Time function: *β*(*s*_*l*_) = *β*_0_ + *β*_1_(*s*_*l*_/10) + *β*_2_(*s*_*l*_/10)^2^, *θ*(*s*_*l*_) = *θ*_1_(*s*_*l*_/10) + *θ*_2_(*s*_*l*_/10)^2^

### Model construction

According to the patients’ median follow-up time of 11.86 years, we defined the  *s*_max_ is 10 year, and selected the prediction interval as [0, 10]. From the completion of kidney transplantation as the initial time point (*s*_0_ = 0) to the 10th year after transplantation (*s*_40_ = 10), we selected 41 landmark time points *s*_*l*_ ∈ {*s*_0_, *s*_1_, …, *s*_40_} in every 3 months. To obtain dynamic survival predictions for the next *w* years, prediction windows of *w* = 5 and *w* = 10 were selected, which are more reasonable for a long-term observation process [23]. The detailed results of the static Cox model and dynamic Cox model (*w* = 5) are depicted in Table 1. We built the static Cox model based on the baseline values of six covariates; however, five covariates did not show significant *P* values (*P* > 0.05). In contrast, the dynamic Cox model showed that age, weight, hematocrit, proteinuria and GFR had significant results. The time function and three time-dependent covariates (hematocrit, proteinuria and GFR) with time functions also had significant *P* values and were included to adjust the model. Taking hematocrit as an example, *β*(*s*_*l*_) = 0.256 − 1.498(*s*_*l*_/10) means that per 0.1% increase in hematocrit, the risk of renal allograft failure increased by 0.256, and as the prediction time *s*_*l*_ increased, the effect of hematocrit decreased. *θ*(*s*_*l*_) = 8.444(*s*_*l*_/10) − 1.957(*s*_*l*_/10)^2^ is the change in the baseline HR *h*_0_(*t*) with increasing prediction time *s*_*l*_.

### Model assessment

**Fig. 2:**
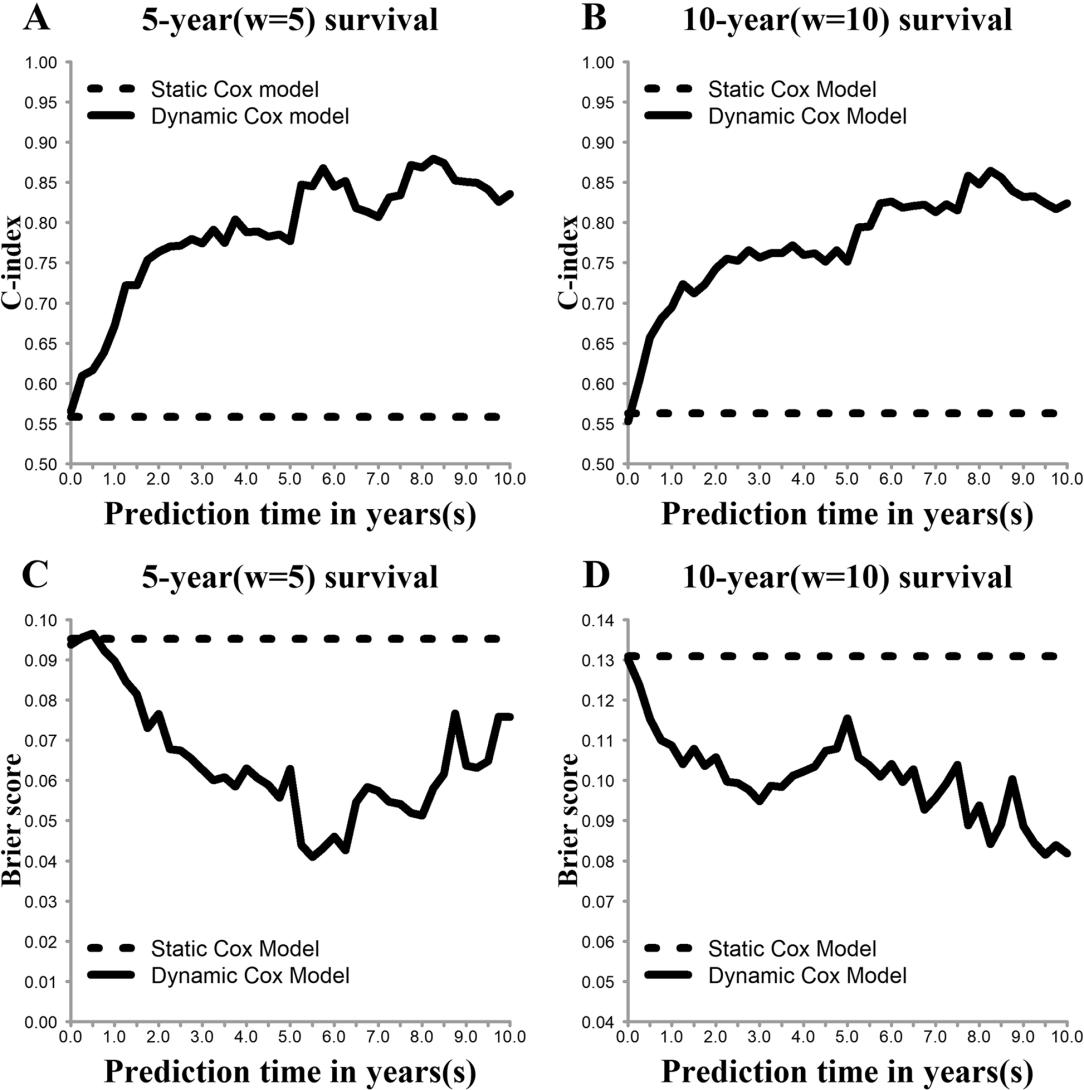
Model assessments (C-indices and Brier scores). Note: A higher C-index indicates a better performing model; a lower Brier score indicates a better performing model

The model assessment measures were calculated according to the 5-year (*w* = 5) and 10-year (*w* = 10) survival rates derived from each landmark time point *s*_*l*_. Figure 2 shows the prediction measures of the two models. We found that compared with the static Cox model, the dynamic Cox model was better in terms of both the C-index and Brier score as the prediction time *s* increased. The changing trends of the C-index and Brier score corresponded to this finding; when the dynamic Cox model had higher discrimination, its calibration was better.

### Dynamic analysis

#### Individual survival prediction

**Fig. 3:**
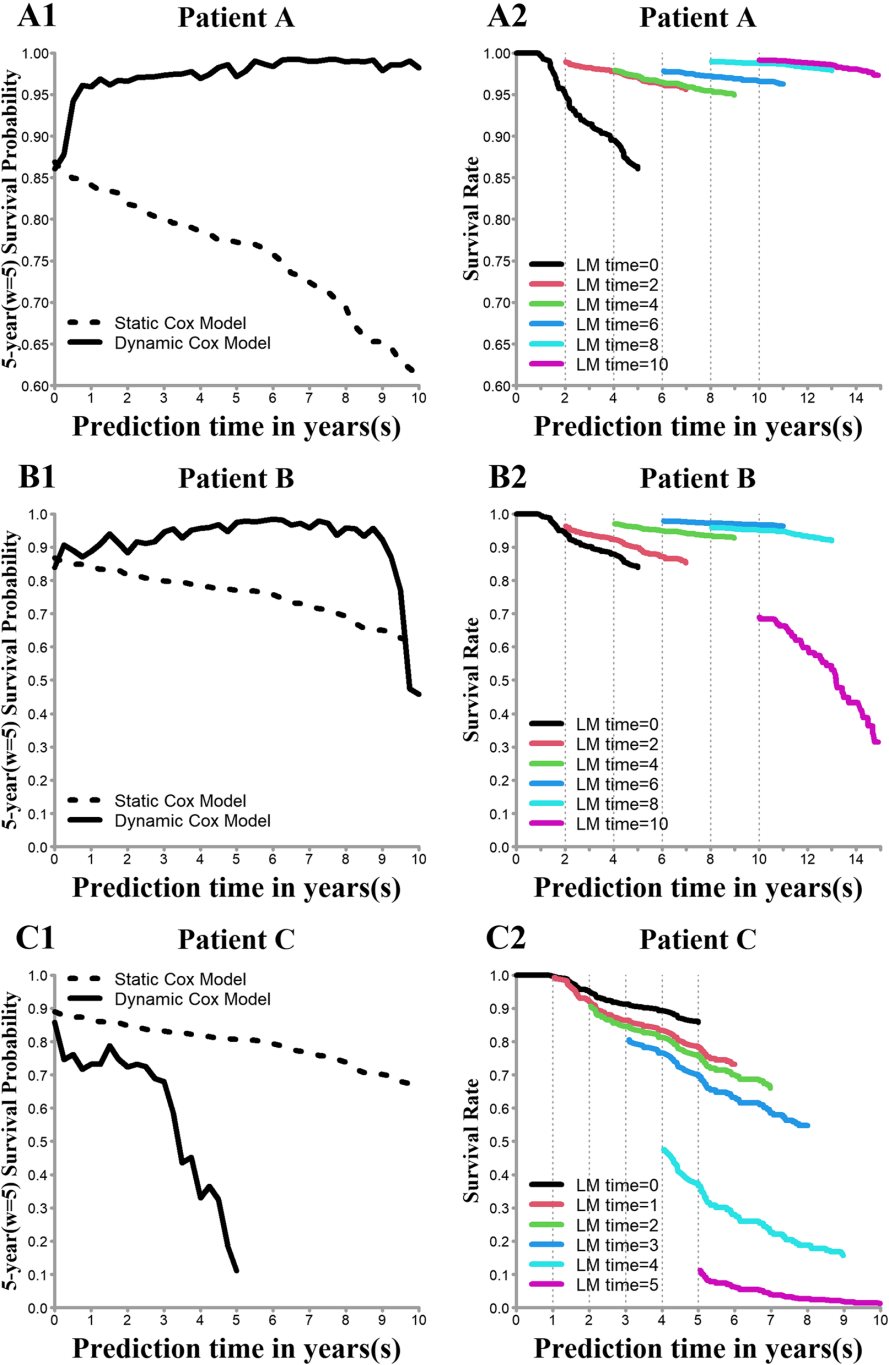
Individual dynamic prediction. Note: A1-C1 show the 5 year conditional survival probability at each prediction time point; A2-C2 show the real-time survival rate from each prediction time point to the next 5 years

Three patients were selected for individual prediction (*w*=5), and detailed information is shown in Supplementary Table S1. The prediction results regarding the individuals are shown in Fig. 3. During the period from kidney transplantation to the first year, as the GFR value of patient A gradually increased and the proteinuria level returned to zero, the conditional survival probability of patient A gradually improved (Fig. 3A1). After the first year, patient A’s condition stabilized, the real-time survival curve gradually tended to be horizontal, and almost all survival rates were greater than 0.95 (Fig. 3A2). In contrast, patient B maintained his condition during 0–7 years of follow-up. However, the situation changed at 7.5 years, when the GFR presented a downward trend, while the proteinuria level gradually increased; at this time, his conditional survival probability declined from over 0.9 to 0.5 (Fig. 3B1), and the survival curve also showed a similar result (Fig. 3B2). The condition of patient C deteriorated from *s* = 0. Figure 3C1-C2 shows that her conditional survival probabilities and real-time survival curves gradually decreased, and patient C died at 5.17 years.

The static Cox model, which only considered the information at baseline (*s* = 0), predicted that the survival probabilities of these patients would gradually decrease over time, and it could not reflect real-time patient survival probabilities with their longitudinal information.

#### Prognostic analysis

**Fig. 4:**
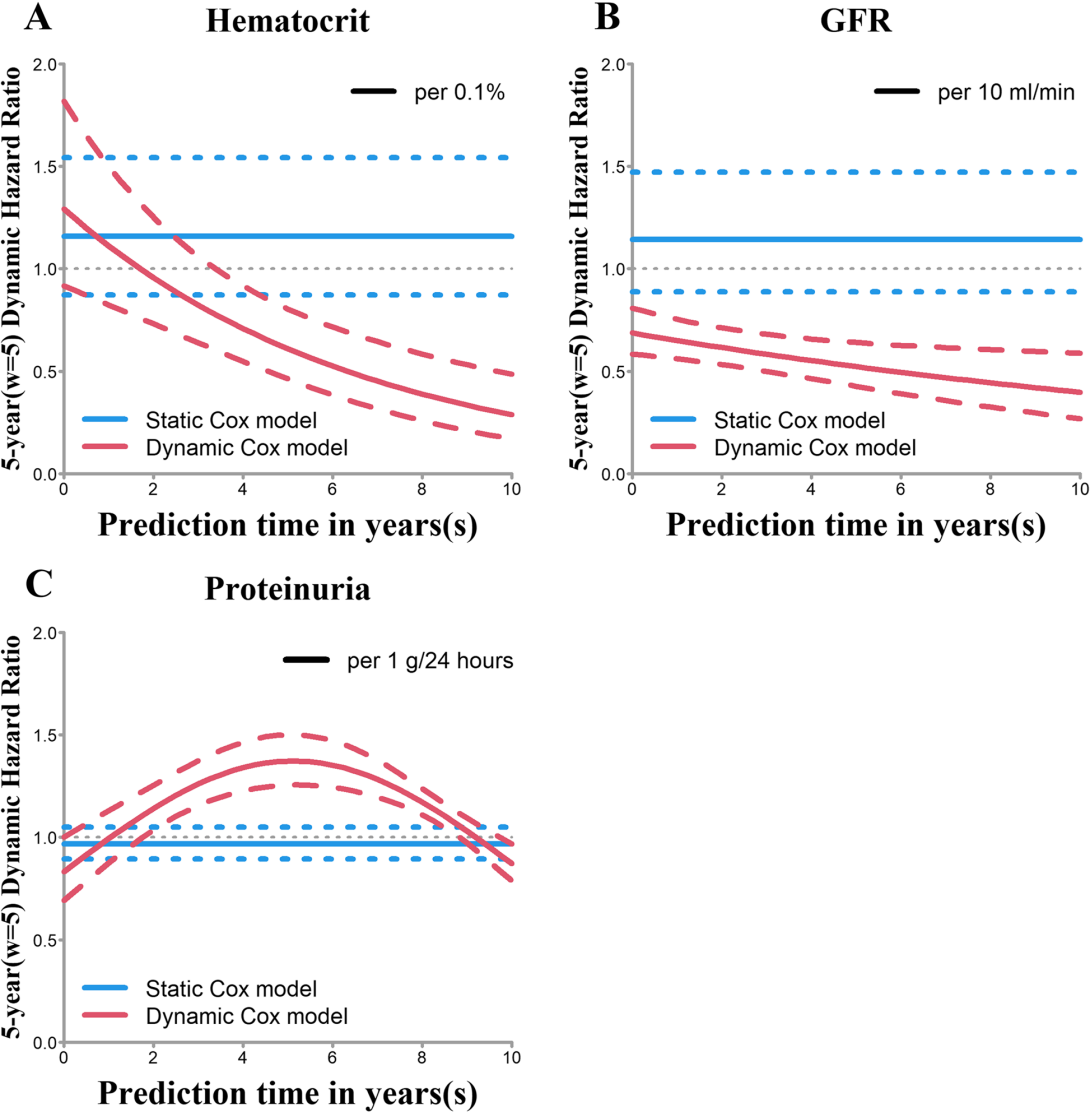
Dynamic HRs and 95% confidence intervals. Note: For example, **A** shows that the dynamic HR of hematocrit at the prediction time point of 0 years (*s* = 0) is 1.292 (hematocrit increase per 0.1%) with no statistical significance (95% confidence intervals includes HR = 1). However, when the prediction time point is 4 years (*s* = 4), the HR changes to 0.736 and is statistically significant (95% confidence intervals excludes HR = 1)

The dynamic Cox model could also be used to conduct a prognostic analysis with the covariates with time-varying effects, while the static Cox model can only obtain the HR from baseline. The dynamic Cox model for predicting 5-year (*w* = 5) survival showed the significance of hematocrit, proteinuria and GFR with time-varying effects (Fig. 4). Taking hematocrit as an example, Fig. 4A shows that the dynamic HR of hematocrit gradually decreased as the prediction time increased and the impact of hematocrit on patients gradually diminished. However, the static Cox model could show only the HR at baseline and with no significant result (95% confidence interval included 0).

Supplementary Appendix S1 illustrates how the static Cox model and the dynamic Cox model can be carried out in practice with the R code.

## Discussion

Precision medicine aims to improve the quality of health care by individualizing the health care process to the uniquely evolving health status of each patient [24]. Dynamic prediction is a method that uses updated follow-up information to predict the individual survival or risk of a disease in real time [25]. Currently, the diagnosis and early treatment of diseases after kidney transplantation are expected to enter an era of individualization. It is important to make dynamic and precise survival predictions for individuals to guide clinical decision-making (such as adjusting immunosuppression, returning to dialysis, or re-enrolling in the transplant waiting list) based on the patients’ longitudinal follow-up information.

Monitoring the survival of renal allografts is a long-term process. During this process, patients want to know the survival probabilities of their allograft at each stage. Patients can learn the condition of their allograft in real time through the use of dynamic prediction models instead of static prediction models. For example, we used the baseline information of kidney transplant patients to build a static prediction model that could only predict the survival of the patients when they entered the study. After a period of time, each patient returned to the hospital for a follow-up, and the values of the covariates were changed. The static prediction model is not suitable for patients who have survived for a period of time after transplantation. In contrast, a dynamic prediction model can use both baseline and follow-up information to predict the survival probabilities of new allografts for patients who have been alive for a period of time after transplantation.

In our study, we selected a landmarking approach to make dynamic predictions that can handle more limitations than joint modeling methods. Many studies have shown that the joint modeling method will have lower predictive performance than the landmarking approach when the model is not specified correctly [26]. Moreover, landmarking the dynamic Cox model can also use the covariates with time-varying effects to make prognoses and predictions [18] at different selected landmark time points and predict the next few years’ survival for patients, so that patients can know their conditions in real time and early treatment can be applied to patients with predicted high risk of allograft failure.

It is indeed challenging for doctors to integrate baseline and posttransplantation clinical information to dynamically predict the risk of renal allograft failure. However, some studies have shown that clinical decisions based on only clinical data and doctors’ judgments are often not accurate and are highly variable between doctors [27]. Therefore, dynamic risk/survival prediction for individuals is important in clinical decision-making. For instance, it may be possible for a patient to consider an earlier return to dialysis or re-enrollment in the transplant waiting list for retransplantation if the predicted risk is very high within a shorter prediction window of an early landmark time point. Thus, when applying the dynamic prediction model based on the landmarking approach, the selection of landmark time points and prediction windows could also be important.

When we apply the landmarking approach, some detailed settings are necessary. First, the prediction window *w* depends on the disease duration or the duration of follow-up. For severe cancers, *w* = 1 or *w* = 2 is relevant, but for some clinical research with a long duration of follow-up, such as that after kidney transplantation, we choose a window of prediction of 5 years as a relevant time horizon to provide middle-term prognoses [28]. Furthermore, the selection of the landmark time point *s*_*l*_ is independent of the actual event time, which implicitly defines the weighting of the prediction time. The simplest method is to use an equidistant grid of points on the prediction interval [*s*_0_, *s*_*L*_] from the time to clinical research entry *s*_0_ to *s*_*L*_, and the number of time points between 20 and 100 is sufficient [23]. In addition, the length of [*s*_0_, *s*_*L*_] may affect the results, usually selecting the time to clinical research entry as *s*_0_ and the median follow-up time as the maximum prediction time of interest *s*_*L*_. For example, in this article, the landmark dataset ***R*** contained only those patients’ longitudinal information from the time point after kidney transplantation to 10 years after transplantation. Finally, the functional form of time-varying effects *β*(*s*) and the baseline hazard changing *θ*(*s*), the most commonly used quadratic functions and spline functions should also be chosen in practice.

There are two limitations to our study. First, existing models for predicting the survival of renal allografts also include predictors such as donor information, primary kidney disease, comorbidities, supportive therapies, and immunization therapies, which we did not include in our model [28, 29]. However, these covariates can always be directly manipulated in clinical practice (subgroups could be made for the analysis), whereas we are more concerned with the impact of directly measured covariates that reflect the changes in renal function on patient graft survival. The GFR and proteinuria used in our study are acknowledged indicators that directly reflect the function of the kidneys, and hematocrit can indirectly reflect the improvement in kidney function and systemic inflammatory conditions [30–32]. The three variables we analyzed were all obtained by measurement and are representative of the outcomes of any intervention. The landmark dynamic Cox model in our study could later gradually incorporate more variables that might be more satisfactory. Second, although the Monte Carlo cross-validation for internal validation of the proposed models performed well in our study, we still strongly encourage external validation, which can assess generalizability in a larger population of kidney recipients. Moreover, the dynamic Cox model constructed from the data presented in our paper can serve as an example, enabling similar types of data in the transplant field to build models for prediction and prognostic analysis.

## Conclusions

In summary, dynamic Cox prediction models can solve the problem that static Cox models can perform “static prediction” only after kidney transplantation, and they can realize dynamic survival prediction by utilizing updated clinical follow-up data. Based on our study, the use of a dynamic Cox model can provide an updated, dynamic and more accurate prediction for patients and may provide evidence and a reference to better guide clinical decision-making for applying early treatment to patients at high risk of allograft failure.

## Supplementary Information

**Additional file 1: Table S1.** Basic information about the patients. **Fig. S1.** Monte Carlo cross validation process. **Fig. S2.** Survival Curve for all patients. **Appendix S1.** R code for building a static Cox model and a dynamic Cox model.

## Data Availability

The dataset supporting the conclusions of this article is extracted from the published article “Rizopoulos D, Ghosh P. A Bayesian semiparametric multivariate joint model for multiple longitudinal outcomes and a time-to-event. Stat Med. 2011;30(12):1366-80. doi: 10.1002/sim.4205” and the data of this article is available publicly from https://gangli.faculty.biostat.ucla.edu/jm-book-data. We obtain the dataset had maintained the anonymity of subjects. We confirm that all methods were carried out in accordance with Declaration of Helsinki.

## Abbreviations

CKD: Chronic kidney disease
GFR: Glomerular filtration rate
HR: Hazard ratio
