# Supplementary material for: Individual dynamic prediction and prognostic analysis for long-term allograft survival after kidney transplantation
Source: BMC Nephrol. 2022 Nov 7;23:359. doi: 10.1186/s12882-022-02996-0 (PMC9641958; doi:10.1186/s12882-022-02996-0)
Supplement: Supplementary file 1 — Additional file 1: Table S1. Basic information about the patients. Fig. S1. Monte Carlo cross validation process. Fig. S2. Survival Curve for all patients. Appendix S1. R code for building a static Cox model and a dynamic Cox model. [file 12882_2022_2996_MOESM1_ESM.docx]

**Supplementary Materials**

**Table S1:** Basic information about the patients.

| **Patient** | **Variables** | | | | | | | | |
| --- | --- | --- | --- | --- | --- | --- | --- | --- | --- |
|  | Surv-T | Status | Age | Weight | Gender | Foll-T | Hema | Prot | GFR |
| A | 18.95 | Censored | 37 | 58.0 | Male | 0.00 | 0.25 | 2.50 | 11.21 |
|  |  |  |  |  |  | 0.25 | 0.29 | 0.00 | 34.49 |
|  |  |  |  |  |  | 0.50 | 0.34 | 0.00 | 58.98 |
|  |  |  |  |  |  | 0.75 | 0.34 | 0.00 | 68.66 |
|  |  |  |  |  |  | 1.00 | 0.34 | 0.00 | 68.52 |
|  |  |  |  |  |  | 1.25 | 0.34 | 0.00 | 74.57 |
| B | 12.13 | Censored | 35 | 56.5 | Male | 7.50 | 0.45 | 0.19 | 46.12 |
|  |  |  |  |  |  | 8.00 | 0.47 | 1.25 | 40.78 |
|  |  |  |  |  |  | 8.50 | 0.46 | 1.58 | 38.28 |
|  |  |  |  |  |  | 9.00 | 0.45 | 1.19 | 34.41 |
|  |  |  |  |  |  | 9.50 | 0.37 | 0.25 | 32.03 |
|  |  |  |  |  |  | 10.00 | 0.34 | 0.00 | 24.87 |
| C | 5.17 | Death | 22 | 60.6 | Female | 0.50 | 0.29 | 0.60 | 38.21 |
|  |  |  |  |  |  | 1.00 | 0.38 | 0.00 | 41.86 |
|  |  |  |  |  |  | 2.00 | 0.31 | 0.00 | 39.53 |
|  |  |  |  |  |  | 3.00 | 0.32 | 0.40 | 37.40 |
|  |  |  |  |  |  | 4.00 | 0.31 | 0.93 | 24.20 |
|  |  |  |  |  |  | 5.00 | 0.31 | 0.80 | 13.04 |

Note: Surv-T, survival time (years); Age, age of transplantation (years old); Weight (kg); Gender (female, male); Foll-T, follow-up time (years); Hema, hematocrit (0.1 %); Prot, proteinuria (g/24 hour); GFR, glomerular filtration rate (ml/min).


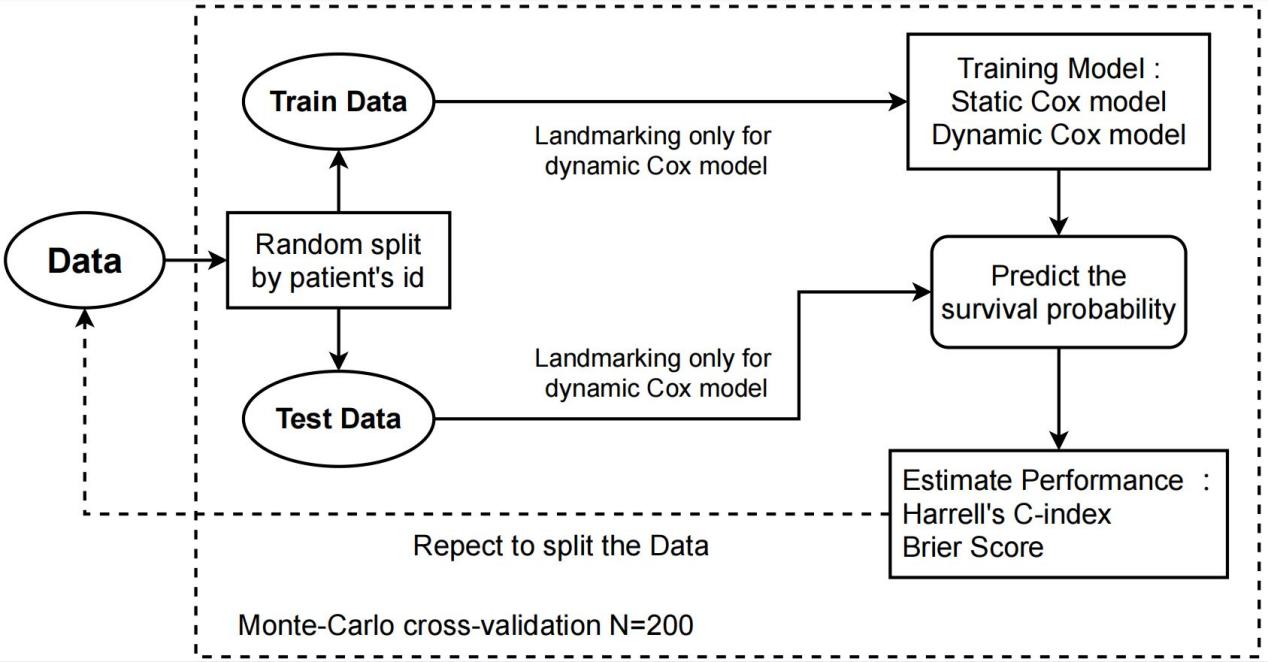


**Figure S1:** Monte Carlo cross validation process.


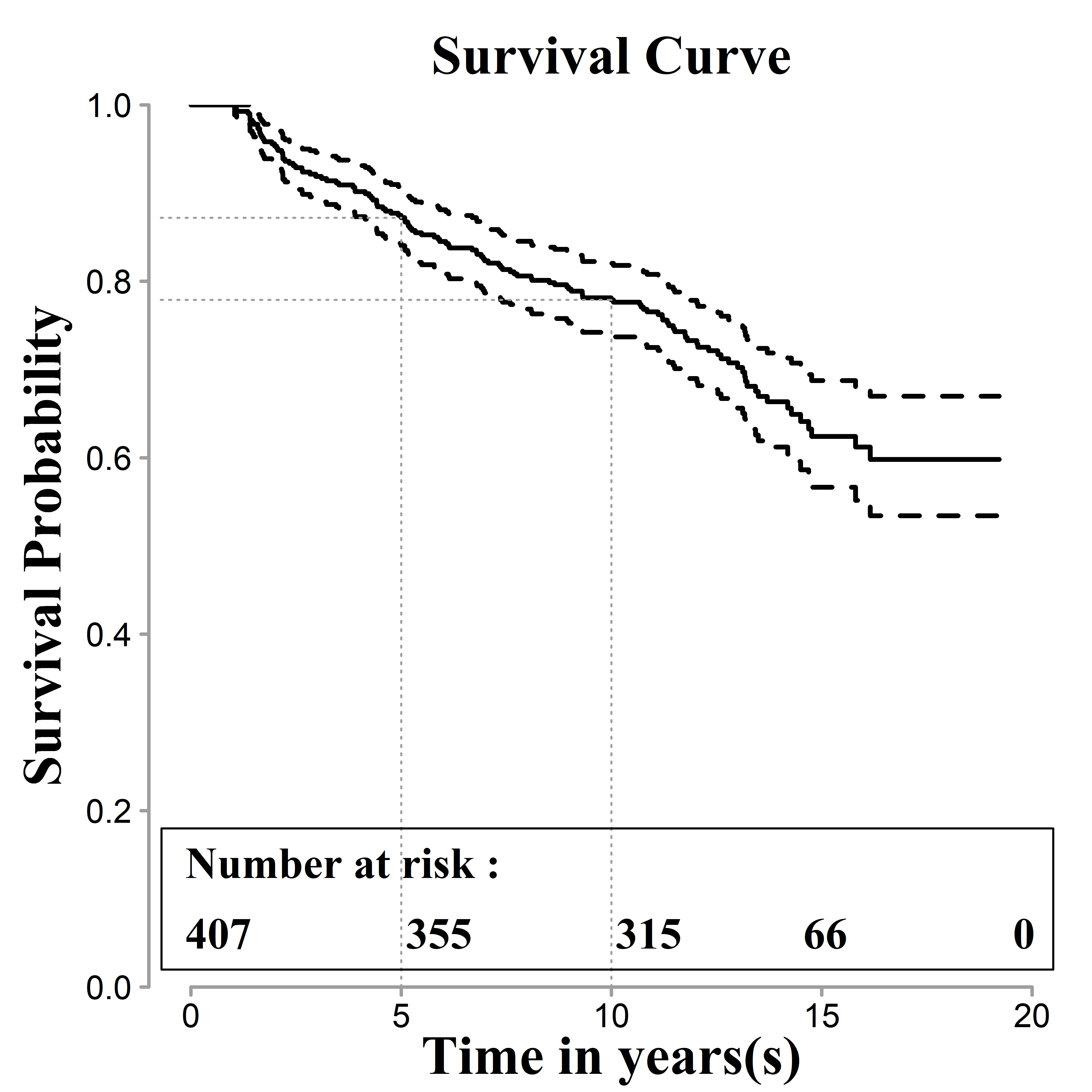


**Figure S2:** Survival Curve for all patients.

**Appendix S1:** R code for building a static Cox model and a dynamic Cox model

In this part, it will be illustrated how the static Cox model and the dynamic Cox model can be carried out in practice with the R software.

Part 1: Introduce the R version and data preparation process.

*### R version 4.0.5 (2021-03-31)
### Platform: x86_64-w64-mingw32/x64 (64-bit)*
*### install the required packages in R
### load the package into memory*
library(sampling)
library(nnet)
library(dynpred)
library(survival)
library(plyr)

*# set your working directory to the location of this file*source("/dynamic_cox_supplement_function.R")

*# Contains all the helper functions called in this file*
*# Due to the data-use agreement, a complete dataset cannot be given.
# Assume that the data file is located on the current working
# directory, it can be read as follows:*data<-read.csv("/data.csv")
head(data)

|  | id | stat | age | sex | weig | hema | gfr | prot | mtime | time |
| --- | --- | --- | --- | --- | --- | --- | --- | --- | --- | --- |
| 1 | 5466 | 1 | 2.5 | 1 | 6.58 | 2.6 | 1.2816734 | 3.8 | 0 | 7.685147 |
| 2 | 5471 | 0 | 4.1 | 1 | 7.31 | 2.5 | 0.9854167 | 5.2 | 0 | 19.219713 |
| 3 | 5472 | 1 | 1.9 | 0 | 4.18 | 3.0 | 0.7193830 | 2.2 | 0 | 8.090349 |
| 4 | 5475 | 0 | 5.5 | 0 | 6.92 | 2.8 | 0.8266700 | 0.8 | 0 | 18.915811 |
| 5 | 5477 | 0 | 3.0 | 1 | 7.72 | 2.8 | 1.2681883 | 3.6 | 0 | 19.011636 |
| 6 | 5479 | 1 | 5.2 | 1 | 7.03 | 1.7 | 1.3018113 | 0.5 | 0 | 12.605065 |

Part 2: Establishment of static Cox model.

*# Obtain the data at baseline*

data2<-data[data$mtime==0,]

formula_cox<- Surv(time,status)~ age+gender+weight+haem+gfr+prot

cox<-coxph(formula_cox,data=data2)

summary(cox)

Part 3: Establishment of dynamic Cox model.
*# The landmark time points sl were established at every six monthes
# between 0 and 10 years after diagnosis*sL<-10
sl<-seq(0,sL,by=0.25)
nsl<-length(sl)
*# the prediction window w=5 years*
w<-5

*# Create a single "super prediction dataset"*
*# Define the type of variables*
fix<-c("age","gender","weight")
vary<-c("hema","gfr","prot")
*# Create the corresponding landmark datasets.*
LMdata<-NULL
for (i in 1:nsl){
 LM<-cutLM(data=data,outcome=list(time="time",status="status"),
 covs=list(fixed=fix,varying=vary),LM=sl[i],horizon=sl[i]+w,
 format="long",id="id",rtime="mtime",right=F)
 LMdata<-rbind(LMdata,LM)
}
*# We define and run a function that generates the interactions*

*between covariates and prediction time the special covariates.*
*# We name this function gen_cov_time.*
gen_cov_time<-function(data,cov,time){
 for (i in 1:length(cov)){
 s<-paste("data$",cov[i],".t",1:2,"<-data$",cov[i],

c("*time","*time^2"),sep="")
 eval(parse(text=s)) }
 data$LM1<-time
 data$LM2<-time^2
 return(data) }

LMdata<-gen_cov_time(data=LMdata,cov=c(fix,vary),

time=LMdata$LM/sL)
head(LMdata)

*# A backward step-down selection process*
formula<-Surv(LM, time, status)~
 age + *#age.t1 + #age.t2 +
 #gender + #gender.t1 + #gender.t2 +* weight + *#weight.t1 + #weight.t2 +* hema + hema.t1 + *#hema.t2 +* gfr + gfr.t1 + *#gfr.t2 +* prot + prot.t1 + prot.t2 +
 LM1 + LM2 +
 cluster(id)

Model<-coxph(formula, data = LMdata, method = "breslow")
output<-(cbind(coef = Model$coefficients,

SE = sqrt(diag(Model$var)),
 PVal = 2-2*pnorm(abs(Model$coefficients/sqrt(diag(Model$var))))
 ))
round(output,3)

Part 4: Model assessment of dynamic Cox model.
*# The Monte-Carlo cross-validation can be used:*
nmc<-500
nbreak<-200
nrun<-0

*# The process for assessing static Cox model*

cindex<-c()

brierscore<-c()

for (i in 1:nmc){

*# The training set contains 70% of the samples.*

*# Remaining 30% is the test set.*

set.seed(20210404+10*i)

index<-sample(x=2,size=length(unique(data2$id)),replace=TRUE,

prob=c(0.7,0.3))

ID<-unique(data2$id)

trainID<-ID[index==1]

testID<-ID[index==2]

traindata<-LMdata2[LMdata2$id %in% trainID,]

testdata<-LMdata2[LMdata2$id %in% testID,]

*# Calculate C-index and Brier score.*

Model<-coxph(formula_cox,data=traindata,method="breslow")

cindex[i]<-cal_cindex(model=Model,data=testdata)

brierscore[i]<-cal_brierscore1(model=Model,traindata,testdata,

width=w,tout=0)

nrun<-nrun+1

if (nrun==nbreak) {break}

}

CI<-mean(cindex)

BS<-mean(brierscore)

*# The process for assessing dynamic Cox model*cindex1<-data.frame(array(,dim=c(nbreak,nsl)))
brierscore1<-data.frame(array(,dim=c(nbreak,nsl)))

for (i in 1:nmc){
 *# The training set contains 70% of the samples.*
 *# Remaining 30% is the test set.*
 set.seed(20210404+10*i)
 index<-sample(x=2,size=length(unique(LMdata$id)),

replace=TRUE, prob=c(0.7,0.3))
 ID<-unique(LMdata$id)
 trainID<-ID[index==1]
 testID<-ID[index==2]
 trainLMdata<-LMdata[LMdata$id %in% trainID,]
 testLMdata<-LMdata[LMdata$id %in% testID,]
 *# Calculate C-index and Brier score for dynamic Cox model.*
 inv<-try(cal_model(formula=formula, trainLMdata, testLMdata),

silent=TRUE)
 if ('try-error' %in% class(inv)){ next }
 else {
 nrun<-nrun+1
 cindex1[nrun,]<-inv[[1]]
 brierscore1[nrun,]<-inv[[2]]
 if (nrun==nbreak) {break}
 }
}
CI1<-apply(cindex1,2,mean)
BS1<-apply(brierscore1,2,mean)

Part 5: Individual dynamic prediction of dynamic Cox model.
*# The id option defines the individual to be predicted*id<-unique(data$id)

*# calculate the conditional survival probability of the Cox Model
# get data2 at baseline*data2<-data[data$mtime==0,]
formula_cox<-Surv(time,status)~ age+gender+weight+hema+gfr+prot
cox<-coxph(formula_cox,data=data2)
cox.sp<-matrix(data = NA, nrow = length(sl), ncol = 2)
colnames(cox.sp)<-c("time",id)
cox.sp[,1]<-sl

survest_cox<-data.frame(time=sl,surv=NA,csurv=NA)
out<-summary(survfit(cox,newdata=data2[which(data2$id==5481),], type="aalen"))
for (i in 1:length(sl)){
 tmp<-evalstep(out$time,out$surv,c(sl[i],sl[i]+w),subst=1)
 survest_cox[i,2]<-tmp[2]
 survest_cox[i,3]<-tmp[2]/tmp[1]
}
cox.sp[,2]<-survest_cox$surv

*# calculate the survival probability of the Dynamic Cox Model*
dc.sp<-matrix(data = NA, nrow = length(sl), ncol = 2)
colnames(dc.sp)<-c("time",id)
dc.sp[,1]<-sl

survest_dc<-data.frame(time=sl,surv=NA)
for (i in 1:length(sl)) {
 dt<-LMdata[which((LMdata$id==5481)&(LMdata$LM==sl[i])),]
 out<-summary(survfit(Model,newdata=dt))
 tmp<-evalstep(out$time,out$surv,c(sl[i],sl[i]+w),subst=1)
 survest_dc[i,2]<-tmp[2]/tmp[1]
}
dc.sp[,2]<-survest_dc$surv

*# get the real-time survival curve*

tt<-data2$time

tt<-sort(unique(c(0,tt,tt-w)))

tt<-tt[tt>=0]

survest_r<-matrix(NA,length(tt),length(sl)+1)

survest_r[,1]<-tt

for (i in 1:length(sl)){

dt<-LMdata[which((LMdata$id==5481)&(LMdata$LM==sl[i])),]

out<-summary(survfit(Model3,newdata=dt))

p<-which(tt>=sl[i] & tt<=(sl[i]+w))

t<-tt[tt>=sl[i] & tt<=(sl[i]+w)]

for (k in 1:length(t) ){

tmp<-evalstep(out$time,out$surv,t[k],subst=1)

survest_r[p[k],i+1]<-tmp

}

}

Part 6: The supporting function for building dynamic Cox model.

*### Function to calculate c-index and Brier score values*
*# "model": the model fitted on the training set*
*# "data": the test set*
cal_cindex1<-function(model,data){
 nt<-length(data$time)
 ord<-order(data$time,-data$status)
 time<-data$time[ord]
 status<-data$status[ord]
 risk<-predict(model,newdata=data,type="lp")[ord]
 wh<-which(status==1)
 total<-con<-0
 for (m in wh){
 for (n in (m:nt)) {
 if (time[n] > time[m]) {
 total <- total + 1
 if (risk[n] < risk[m])
 con <- con + 1
 if (risk[n] == risk[m])
 con <- con + 0.5 } } }
 return(con/total)
}

*# "model": the model fitted on the training set*
*# "Tdata": the training set*
*# "Vdata": the test set*
*# "width": the prediction window*
*# "tout": the prediction time*
cal_brierscore1<-function(model,Tdata,Vdata,width,tout) {
 ord<-order(Vdata$time,-Vdata$status)
 time<-Vdata$time[ord]
 status<-Vdata$status[ord]
 risk<-predict(model,newdata=Vdata,type="lp")[ord]
 cox1<-coxph(Surv(Vdata$LM[ord],Vdata$time[ord],

Vdata$status[ord])~risk)
 if (sum(risk^2)==0)
 sf<-survfit(cox1,newdata=data.frame(risk=risk),type="kalbfl")
 else sf<-survfit(cox1,newdata=data.frame(risk=risk))
 tt<-sf$time
 survmat<-sf$surv
 if (tt[1]>0){
 tsurv<-c(0,tt)
 survmat<-rbind(rep(1,nrow(Vdata)),survmat)
 }
 else tsurv<-tt
 coxcens<-coxph(Surv(LM,time,1-status)~1,data=Tdata)
 xcens<-predict(coxcens,newdata=Vdata,type="lp")[ord]
 coxcens<-coxph(Surv(Vdata$LM,Vdata$time,1-Vdata$status)~xcens)
 if (sum(xcens^2)==0)
 sfcens<-survfit(coxcens,newdata=data.frame(xcens=xcens),

type="kalbfl")
 else sfcens<-survfit(coxcens,newdata=data.frame(xcens=xcens))
 tcens<-sfcens$time
 censmat<-sfcens$surv
 if (tcens[1]>0) {
 tcens<-c(0,tcens)
 censmat<-rbind(rep(1,nrow(Vdata)),censmat)
 }
 res<-pew(time,status,tsurv,survmat,tcens,censmat,

width,"Brier",tout)
 return(res[1,2])
}

*# Performance measures (Harrell’s C-index and Brier score) were*
*# obtained for each landmark time point (Model 3)*
cal_model<-function(formula,TSet,VSet){
 cindex<-rep(NA,nsl)
 score<-rep(NA,nsl)
 Model<-coxph(formula,data=TSet,method="breslow")
 for (i in 1:nsl){
 Vdata<-VSet[VSet$LM==sl[i],]
 cindex[i]<-cal_cindex(model=Model,data=Vdata)
 score[i]<-cal_brierscore(model=Model,TSet,Vdata,

width=w,tout=sl[i])
 }
 return(list(cindex=cindex,brierscore=score))
}

----------------------------------

**Plot making process**

----------------------------------

Part 1: Plot dynamic hazard ratio curves with 95% CI (dynamic Cox model).
*# variable=hema*

hema_hr=1.1596

hema_hr1=0.8714

hema_hr2=1.5433
bet<-matrix(c(rep(1,length(sl)),sl/sL),length(sl),2)

d_HR<-data.frame(sl=sl,logHR=as.numeric(bet %*% Model3$coef[3:4]))

se<-sqrt(diag(bet %*% Model3$var[3:4,3:4] %*% t(bet)))

d_HR$lower<-exp(d_HR$logHR-qnorm(0.975)*se)

d_HR$upper<-exp(d_HR$logHR+qnorm(0.975)*se)

tiff(file="/Figure 4-A1.tif",width=4750,height=4750,res=300, pointsize=23.5,compression="lzw")

plot(x=NULL,xlim=c(0,sL),ylim=c(0,2),bty="n",xaxt="n",yaxt="n")

axis(1,las=1,pos=0,cex.axis=1.5,tcl=-0.3,hadj=0.5,padj=-0.3,

lwd=5,col=8)

axis(2,las=1,pos=0,cex.axis=1.5,tcl=-0.3,hadj=0.7,padj=0.5,

lwd=5,col=8)

lines(d_HR$sl,rep(1,41),type="l",lty=3,lwd=5,col=8)

lines(d_HR$sl,rep(hema_hr,41),type="l",lty=1,lwd=10,col=4)

lines(d_HR$sl,rep(hema_hr1,41),type="l",lty=3,lwd=10,col=4)

lines(d_HR$sl,rep(hema_hr2,41),type="l",lty=3,lwd=10,col=4)

lines(d_HR$sl,exp(d_HR$logHR),type="l",lty=1,lwd=10,col=2)

lines(d_HR$sl,d_HR$lower,type="l",lty=2,lwd=10,col=2)

lines(d_HR$sl,d_HR$upper,type="l",lty=2,lwd=10,col=2)

legend(0,0.4,c("Static Cox model","Dynamic Cox model"),

col=c(4,2),lwd=10,bty="n",xpd=TRUE,cex=2,lty=1)

title(main=list("A1 ",font=7,cex=6),line=0.5)

title(main="Haematocrit",font.main=7,cex.main=3,line=1.2)

title(xlab="Prediction time in years(s)",

font.lab=7,cex.lab=3,line=2.5)

title(ylab="5-year(w=5) Dynamic Hazard Ratio",

font.lab=7,cex.lab=2.3,line=1.8)

dev.off()

Part 2: Make the C-index and Brier score plot.

*### C-index plot*

tiff(file="/Figure 2-A.tif",

width=4750,height=4750,res=300, pointsize=23.5,compression="lzw")

plot(x=NULL, xlim=c(0,10),ylim=c(0.5,1),xaxt="n",yaxt="n",bty="n",

xlab='',ylab='',type="l")

axis(1,at=seq(0,10,0.5),las=1,pos=0.5,cex.axis=1.5,tcl=-0.3,padj=-0.3,lwd=5,col=8)

axis(2,at=seq(0.5,1,0.05),las=1,pos=0,cex.axis=1.5,tcl=-0.3,padj=0.5,lwd=5,col=8)

lines(sl,CI1,lwd=16,col=1,lty=1)

lines(sl,CI,lwd=16,col=1,lty=3)

legend(0,1,c("Static Cox model","Dynamic Cox model"),

col=1,lwd=16,bty="n",xpd=TRUE,cex=2,lty=c(3,1))

title(main=list("A ",font=7,cex=6),line=0.5)

title(main="5-year(w=5) survival",font.main=7,cex.main=3,line=1.2)

title(xlab="Prediction time in years(s)",

font.lab=7,cex.lab=3,line=2.5)

title(ylab="C-index",font.lab=7,cex.lab=2.5,line=2.3)

dev.off()

*### Brier score plot*

tiff(file="/Figure 2-C.tif",

width=4750,height=4750,res=300, pointsize=23.5,compression="lzw")

plot(x=NULL,xlim=c(0,10),ylim=c(0,0.1),xaxt="n",yaxt="n",bty="n",

xlab='',ylab='',type="l")

axis(1,at=seq(0,10,0.5),las=1,pos=0,cex.axis=1.5,tcl=-0.3,padj=-0.3,

lwd=5,col=8)

axis(2,at=seq(0,0.1,0.01),las=1,pos=0,cex.axis=1.5,tcl=-0.3,padj=0.5,lwd=5,col=8)

lines(sl,BS1,lwd=16,col=1,lty=1)

lines(sl,BS,lwd=16,col=1,lty=3)

legend(0,0.025,c("Static Cox Model","Dynamic Cox Model"),

col=1,lwd=16,bty="n",xpd=TRUE,cex=2,lty=c(3,1))

title(main=list("C ",font=7,cex=6),line=0.5)

title(main="5-year(w=5) survival",font.main=7,cex.main=3,line=1.2)

title(xlab="Prediction time in years(s)",

font.lab=7,cex.lab=3,line=2.5)

title(ylab="Brier score",font.lab=7,cex.lab=2.5,line=2.3)

dev.off()

Part 3: Make the individual dynamic prediction plot.

*# the conditional survival probability*

tiff(file="/Figure 31-5481.tif",width=4750,height=4750,res=300,

pointsize=23.5,compression="lzw")

plot(x=NULL,type="l",xlim=c(0,sL),ylim=c(0.6,1),bty="n",xaxt="n",

yaxt="n",xlab="",ylab="")

legend(0,0.7,c("Static Cox Model","Dynamic Cox Model"), bty="n",

lty=c(3,1),lwd=12,col=1,xpd=TRUE,cex=1.8)

axis(1,at=seq(0,10,1),las=1,pos=0.6,cex.axis=1.5,tcl=-0.3,hadj=0.5,

padj=-0.3,lwd=5,col=8)

axis(2,at=seq(0.6,1,0.05),las=1,pos=0,cex.axis=1.5,tcl=-0.3,hadj=0.8,

padj=0.5,lwd=5,col=8)

lines(sl,cox.sp,lwd=12,lty=3,col=1)

lines(sl,dc.sp,lwd=12,lty=1,col=1)

title(main=list("A1 ",font=7,cex=6),line=0.5)

title(main="Patient A",font.main=7,cex.main=3,line=1.2)

title(xlab="Prediction time in years(s)",

font.lab=7,cex.lab=3,line=2.5)

title(ylab="5-year(w=5) Survival Probability",

font.lab=7,cex.lab=2.2,line=2)

dev.off()

*# the real-time survival curve*

tiff(file="/Figure 32-5481.tif",width=4750,height=4750,res=300,

pointsize=23.5,compression="lzw")

plot(x=NULL,xlim=c(0,15),ylim=c(0.6,1),bty="n",xaxt="n",yaxt="n",

xlab="",ylab="")

axis(1,labels=F,at=seq(0,15,1),las=1,pos=0.6,cex.axis=1.5,tcl=-0.3,

hadj=0.5,padj=-0.3,lwd=5,col=8)

axis(1,at=seq(0,15,2),las=1,pos=0.6,cex.axis=1.5,tcl=-0.3,hadj=0.5,

padj=-0.3,lwd=5,col=8)

axis(2,at=seq(0.6,1,0.05),las=1,pos=0,cex.axis=1.5,tcl=-0.3,hadj=0.8,padj=0.5,lwd=5,col=8)

lines(rep(2,2),c(0.6,1),type="l",lwd=2,lty=3,col=8)

lines(rep(4,2),c(0.6,1),type="l",lwd=2,lty=3,col=8)

lines(rep(6,2),c(0.6,1),type="l",lwd=2,lty=3,col=8)

lines(rep(8,2),c(0.6,1),type="l",lwd=2,lty=3,col=8)

lines(rep(10,2),c(0.6,1),type="l",lwd=2,lty=3,col=8)

lines(survest_r[,1],survest_r[,2],type="l",lwd=12,lty=1,col=1)

lines(survest_r[,1],survest_r[,10],type="l",lwd=12,lty=1,col=2)

lines(survest_r[,1],survest_r[,18],type="l",lwd=12,lty=1,col=3)

lines(survest_r[,1],survest_r[,26],type="l",lwd=12,lty=1,col=4)

lines(survest_r[,1],survest_r[,34],type="l",lwd=12,lty=1,col=5)

lines(survest_r[,1],survest_r[,42],type="l",lwd=12,lty=1,col=6)

legend(0,0.77,c("LM time=0","LM time=2","LM time=4","LM time=6","LM time=8","LM time=10"),bty="n",lty=1,lwd=12,col=c(1:6),xpd=TRUE,cex=1.8)

title(main=list("A2 ",font=7,cex=6),line=0.5)

title(main="Patient A",font.main=7,cex.main=3,line=1.2)

title(xlab="Prediction time in years(s)",

font.lab=7,cex.lab=3,line=2.5)

title(ylab="5-year(w=5) Survival Rate",font.lab=7,cex.lab=2.2,line=2)

dev.off()
